# Temporal patterns of cortical proliferation of glial cell populations after traumatic brain injury in mice

**DOI:** 10.1042/AN20130034

**Published:** 2014-05-08

**Authors:** Bala T.S. Susarla, Sonia Villapol, Jae-Hyuk Yi, Herbert M. Geller, Aviva J. Symes

**Affiliations:** *Center for Neuroscience and Regenerative Medicine, Uniformed Services University of the Health Sciences, Bethesda, MD, U.S.A.; †Department of Pharmacology, Uniformed Services University of the Health Sciences, Bethesda, MD, U.S.A.; ‡Developmental Neurobiology Section, Division of Intramural Research, National Heart, Lung, and Blood Institute, National Institutes of Health, Bethesda, MD, U.S.A.

**Keywords:** astrocytes, BrdU, microglia, neurogenesis, NG2, proliferation, traumatic brain injury (TBI), BrdU, bromodeoxyuridine, CCI, controlled cortical impact, CNS, central nervous system, DCX, doublecortin, dpi, days post-injury, GFAP, glial fibrillary acidic protein, OPC, oligodendrocyte progenitor cell, PFA, paraformaldehyde, SVZ, subventricular zone, TBI, traumatic brain injury

## Abstract

TBI (traumatic brain injury) triggers an inflammatory cascade, gliosis and cell proliferation following cell death in the pericontusional area and surrounding the site of injury. In order to better understand the proliferative response following CCI (controlled cortical impact) injury, we systematically analyzed the phenotype of dividing cells at several time points post-lesion. C57BL/6 mice were subjected to mild to moderate CCI over the left sensory motor cortex. At different time points following injury, mice were injected with BrdU (bromodeoxyuridine) four times at 3-h intervals and then killed. The greatest number of proliferating cells in the pericontusional region was detected at 3 dpi (days post-injury). At 1 dpi, NG2^+^ cells were the most proliferative population, and at 3 and 7 dpi the Iba-1^+^ microglial cells were proliferating more. A smaller, but significant number of GFAP^+^ (glial fibrillary acidic protein) astrocytes proliferated at all three time points. Interestingly, at 3 dpi we found a small number of proliferating neuroblasts [DCX^+^ (doublecortin)] in the injured cortex. To determine the cell fate of proliferative cells, mice were injected four times with BrdU at 3 dpi and killed at 28 dpi. Approximately 70% of proliferative cells observed at 28 dpi were GFAP^+^ astrocytes. In conclusion, our data suggest that the specific glial cell types respond differentially to injury, suggesting that each cell type responds to a specific pattern of growth factor stimulation at each time point after injury.

## INTRODUCTION

Injuries to the CNS (central nervous system), including TBI (traumatic brain injury), are accompanied by a rapid reactive response that results in the formation of a glial scar around the injury area (Fawcett & Asher, 1999). This scar is comprised of several different cellular elements, including astrocytes, microglia, OPCs (oligodendrocyte progenitor cells) and fibroblasts (Tan et al., 2005; Sofroniew & Vinters, 2010; Kawano et al., 2012). While the ostensible function of the glial scar is to wall off the injury area from further damage (Bush et al., 1999), cells in the scar tissue elaborate a complex extracellular matrix that also prevents regrowth of injured axons and migration of other cells into and out of the injured area (Fawcett & Asher, 1999; Silver & Miller, 2004; Carulli et al., 2005). Thus the role of the glial scar after injury is both beneficial and detrimental.

In the cortical region surrounding the lesion, that will form the glial scar there is significant glial proliferation after injury (Liu et al., 2000). In some injury models, astrogliosis can occur with hypertrophy but little evidence of cell division (Qu & Jakobs, 2013), whereas other models are characterized by a significant number of astrocytes that display mitotic markers (Chirumamilla et al., 2002; Alonso, 2005; Buffo et al., 2008). Moreover, whether mature astrocytes reenter the cell cycle, or other progenitor cells differentiate into astrocytes is still a subject of debate. Proliferating astrocytes are critical to the recovery from severe traumatic injury as transgenic experiments that ablated proliferating astrocytes after injury resulted in larger lesion, greater penetration of infiltrating immune cells and a slower reformation of the blood–brain barrier, in addition to reducing scar formation (Bush et al., 1999). NG2^+^ glia proliferative significantly in response to CNS injury (Rhodes et al., 2006). While initially considered to be oligodendrocyte precursors alone, genetic fate mapping studies have showed that these NG2^+^ cells may show some lineage plasticity after injury to occasionally become astrocytes or myelinating Schwann cells (Zhao et al., 2009). NG2^+^ cells also migrate and become emeshed in the glial scar to contribute to the deposition of the extracellular matrix that prevents regrowth of injured axons (Rhodes et al., 2003). Proliferation is also a part of the microglial response to injury (Rhodes et al., 2003). Microglia rapidly become hypertrophic and ameboid after injury (Giulian, 1987), secreting many pro-inflammatory molecules that can regulate the behavior of other glial cells and have a detrimental effect on neuronal survival (Marin-Teva et al., 2011; Benarroch, 2013). Indeed, pharmacological inhibition of microglial activation and proliferation is a potential therapy for acute brain injury (Homsi et al., 2010).

Although several previous studies have described various cellular proliferative responses in neurogenic regions following injury (Chirumamilla et al., 2002; Bye et al., 2011), the cortical proliferative response after CCI (controlled cortical impact) injury in mouse has not completely been characterized (Kernie et al., 2001; Ramaswamy et al., 2005a). Progenitor cells that reside in the neurogenic niches increase their proliferation after TBI (Bye et al., 2011). While cells in the hippocampal dentate gyrus increase their proliferation with little change in the eventual cell fate of the progenitors (Parent, 2003; Qiu et al., 2007), progenitors in the SVZ (subventricular zone) of the lateral ventricle increase their proliferation and alter their migration and eventual cell fate (Gotz & Huttner, 2005). After injury to the cerebral cortex, progenitors from the SVZ migrate toward the cortical injury, and differentiate usually into glial cells around the injury site (Chirumamilla et al., 2002; Ramaswamy et al., 2005a). These glial cells display unique activation phenotypes responding to the many signaling cascades activated during the secondary injury response (Tatsumi et al., 2005). For these reasons, understanding the temporal pattern of proliferation following injury can contribute to the design of repair strategies.

In this work, we have examined the time course of proliferation of the major classes of reactive cells soon after a CCI injury in the mouse brain. We used a BrdU (bromodeoxyuridine) injection protocol designed to label the entire proliferating faction during the first week following injury combined with immunocytochemistry to identify the cell types that incorporate BrdU. Our results demonstrate a characteristic pattern of cell proliferation for each cell type, both neuronal and glial. In addition, we demonstrate that the proliferation continues well after the first week after injury.

## MATERIALS and METHODS

### Animals

All animal studies were approved by the USUHS Institutional Animal Care and Use committee and were conducted in accordance with the NRC guide to the Care and Use of Laboratory Animals. The 9–10-week-old male C57BL/6 mice (NCI, Frederick, MD) were housed in the regular cages with access to food and water *ad libitum* and a 12:12 light/dark cycle. Mice were allowed to acclimatize to the animal facilities for several days after arrival.

### CCI injury

Mice were anesthetized with isoflurane (4% for induction, 2–3% for maintenance) and securely positioned in a mouse stereotaxic frame (Stoelting Co). Surgery was performed as described previously (Villapol et al., 2012; Yi et al., 2012). Briefly, an incision was made over the forehead, and the scalp was reflected to expose the skull. A craniotomy was made over the left hemisphere and the bone flap was carefully removed. Mice were injured over the left somatosensory cortex (0 bregma, 2 mm lateral to the suture line) at an impact depth of 1 mm with a 2-mm diameter round impact tip (speed 3.6 m/s, dwell time 100 ms) using an electromagnetically driven CCI injury device (Impact One™ stereotaxic impactor CCI, Leica Microsystems GMBH) (Brody et al., 2007; Pleasant et al., 2011). These CCI parameters lead to an injury that is considered mild to moderate according to our experience and previous publications (Washington et al., 2012; Yi et al., 2012). The dura remained intact following craniotomy. Impact caused occasional extradural hemorrhages with mild edema. Following injury, the bone flap was replaced but not secured, and the scalp was sutured closed. Mice were under isoflurane for no longer than 15 min. After recovery from anesthesia, mice were maintained in a warm recovery cage for 1 h and returned to home cages.

### BrdU injection

BrdU (Sigma) was dissolved in 0.9% (w/v) NaCl at a concentration of 10 mg/ml. In order to label all the proliferative cells at any one time point, all mice received a total of 4 i.p. (intraperitoneal) injections spaced at 3 h intervals. Thus, the final injection was 9 h after the initial one. Three groups of mice received their first injection of BrdU (100 mg/kg) at 24, 72 or 168 h following injury and were killed 30 min after the last injection of BrdU. Therefore the time points of killing were at 33.5, 81 and 177.5 h post-injury. We refer to these killing times as 1, 3 and 7 dpi (days post-injury) for simplification. To determine the fate of proliferative cells the fourth group of mice were injected with BrdU on day 3, starting at 72 h after injury with the same protocol, and killed on day 28 after injury.

### Preparation of tissue

Mice were deeply anesthetized with ketamine/xylazine and transcardially perfused with PBS followed by 4% (w/v) PFA (paraformaldehyde). Brains were dissected and post-fixed overnight in 4% PFA, and then transferred to 30% (w/v) sucrose solution stored at 4°C for at least 48 h. Approximately 30-μm-thick serial sections were cut using a microtome (Leica SM 2010R) connected to a freezing stage (Physitemp Inc, BFS-30 MP Controller). All sections were collected sequentially in 96-well plates and stored in antifreeze solution [30% (w/v) glucose, 30% (v/v) ethylene glycol and 1% (v/v) polyvinypyrrolidone in 0.01 M phosphate buffer] at −20°C until use. Free-floating brain sections were used for immunohistochemical staining.

### Immunohistochemistry

For BrdU staining, all sections were washed with PBS three times, denatured (2 N HCl) for 1 h, neutralized with 0.1 M boric acid, pH 8.5 for 20 min and washed with PBS three more times. Sections were then blocked in 10% (v/v) NGS (normal goat serum)/0.5% (v/v) Triton X-100/1X PBS for 1 h before incubation with rat anti-BrdU (1:200; Accurate), with or without cell-specific antisera for 36–48 h at 4°C. The following antisera against cell-specific markers were used: rabbit anti-NG2 (1:400, Millipore), rabbit anti-GFAP (glial fibrillary acidic protein) (1:1000, DAKO), rabbit anti-Iba-1 (1:400, WAKO) rabbit anti-CD11b (1:200, Serotec), mouse anti-S100β (1:500, Sigma) and rabbit anti-DCX (doublecortin) (1:200, Santa Cruz). Sections were washed three times in PBS and incubated with the corresponding Alexa Fluor 488 or 568-conjugated IgG secondary antibodies (all 1:100; Jackson Immunoresearch) for 1 h at room temperature. Sections were rinsed with PBS, mounted on to the slides and coverslipped with ProLong Gold antifade reagent with DAPI (4′,6-diamidino-2-phenylindole) (Invitrogen).

### Quantitative analysis

Three to six animals were analyzed at each time point after the injury for either sham or CCI groups. Three 30-μm-thick coronal sections per brain were selected, spaced 210 μm apart across the region of interest in each animal (from Bregma +0.7 mm to Bregma −0.7 mm). To show consistency of lesion size between animals, an average injury score was calculated for mice at each time point (Supplementary Table S1 at http://www.asnneuro.org/an/006/an006e143add.htm). A systematic stereologic approach was used to count BrdU^+^ cells in the perilesional cortex. To count the total number of BrdU^+^ cells, three fields were selected in the perilesional area with the 10× objective. For counting the cell-specific proliferative cells, five fields per brain section in the pericontusional cortical region were counted under the 40× objective. Cell counts were performed using ImageJ software (National Institutes of Health), compiled and analyzed. Counting was performed by a blinded observer. Images were acquired on an Olympus BX61 with attached Imaging Retiga EXi Aqua CCD camera, and iVision software (BioVision Technologies). A confocal microscope (Zeiss LSM 510) was used to take images from double-labeled brain sections.

## RESULTS

### Cortical cell proliferation corresponds with spatial–temporal patterns in the injured cortex

To determine the timing of the glial cell proliferative response in response to mild-to-moderate CCI injury, we injected mice with the mitotic marker BrdU using a saturation protocol to ensure that almost all dividing cells at any single time point would be labeled with BrdU (Cao et al., 1999). We used time points of 1 dpi to capture the early phase of inflammation, 3 dpi to determine proliferation when astrocytes begin to be activated and 7 dpi to investigate the proliferation as the acute phase is over but when longer term changes may be beginning.

**Figure 1:**
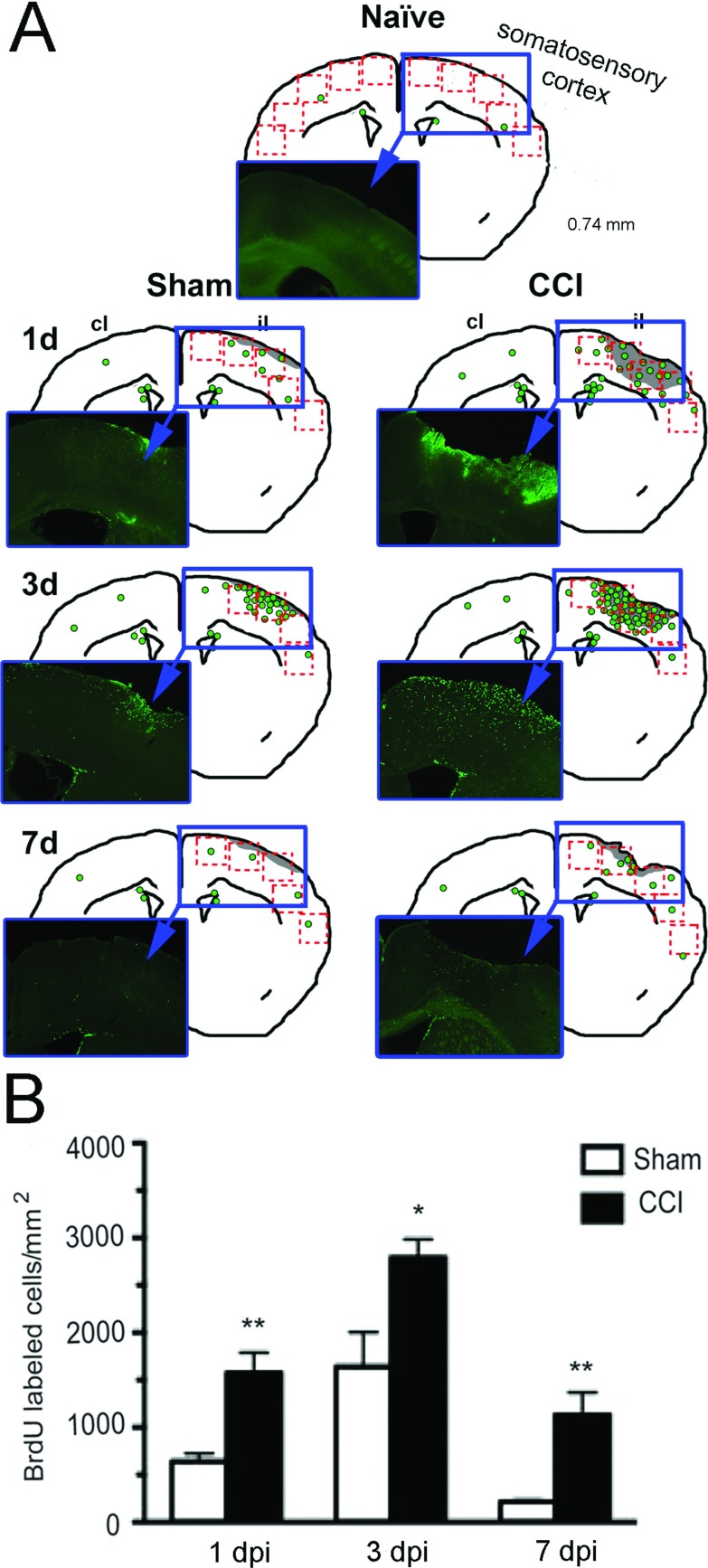
Time course of cortical cell proliferation after CCI injury (A) Representative drawings of a mid-brain section (at +0.74 mm from Bregma) indicating the distribution of BrdU^+^ cells (green circles) in coronal sections of mouse brain at different time points after sham or CCI injury to the somatosensory cortex. The lesion in the right cortex is shown in light gray. The red boxes indicate the five contiguous cortical fields where BrdU^+^ cells were counted. A representative photomicrograph (within blue insets) show actual BrdU^+^ staining. (B) Quantification of total number of BrdU^+^ cells around the cortical injury site in mouse brain after sham or CCI at 1, 3 or 7 dpi. A significant increase in BrdU-labeled cells in the CCI-injured mice compared with sham mice was found at all time points with a peak at 3 dpi (mean±S.E.M., *n*=3–6,*P<0.05; **P<0.01). CCI, controlled cortical impact injury; il, ipsilateral hemisphere; cl, contralateral hemisphere.

BrdU^+^ cells were most pronounced in the pericontusion region (Figure 1A). BrdU^+^ cells were also detected throughout the white matter tracts lining the ipsilateral wall of the lateral ventricle and extending into the corpus callosum, hippocampus and cortex. Significant numbers of BrdU^+^ cells were detected in the cortex in the pericontusion region as early as 1 dpi, with the greatest number of BrdU^+^ cells detected at 3 dpi, with fewer BrdU^+^ cells detected by 7 dpi (Figure 1B). There were also significant numbers of BrdU^+^ cells in mice that had undergone sham procedures indicating that sham surgery was similar to a mild injury (Figure 1B). However, in the naive mouse cortex, there were negligible BrdU^+^ cells (Figure 1A). As the BrdU injection protocol labels the vast majority of dividing cells at any single time point, our data suggest that injury-induced cell proliferation is greatest about 3 dpi.

### Activated microglia are the most proliferative cell population at 3 dpi

**Figure 2:**
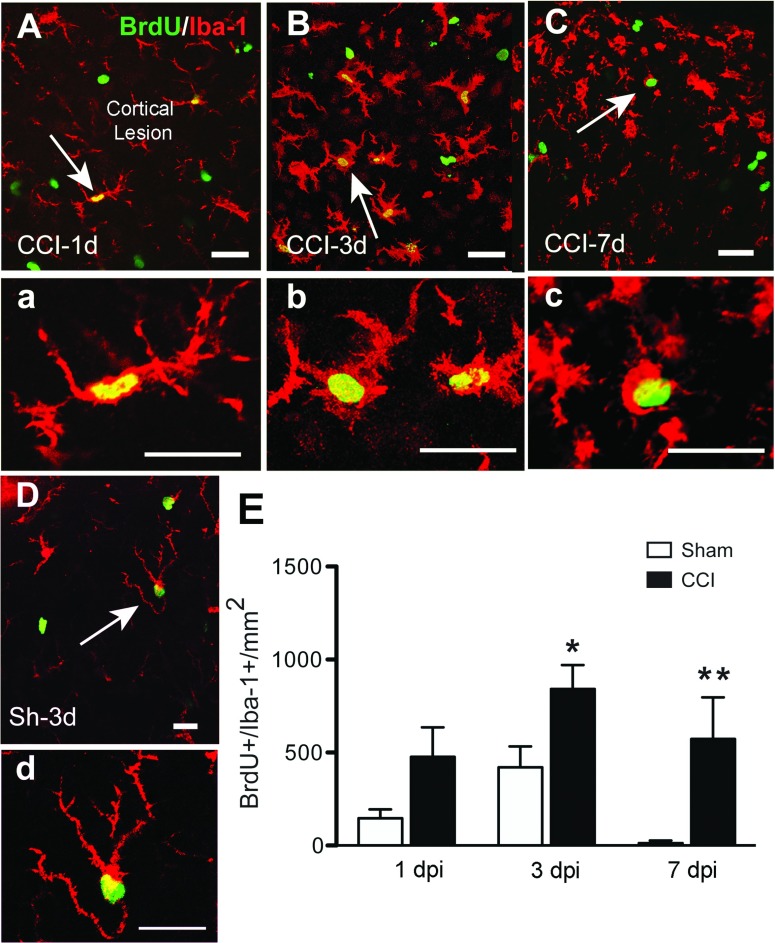
Microglial proliferation after CCI injury Confocal images show localization of proliferating microglial cells (Iba-1^+^, red, BrdU^+^, green) in the perilesional area of the cortex at 1, 3 and 7 dpi. At 1 dpi Iba1^+^ /BrdU^+^ cells have activated microglial morphology (A, inset in a). By 3 dpi proliferating amoeboid microglial cells showed hypertrophic cell body and shorter, thicker ramifications (B, inset in b). There is still significant proliferation of microglial cells at 7 dpi, with cells exhibiting a macrophage-like morphology (C, inset in c). In sham mice, proliferating microglia in cortical areas show a highly ramified phenotype, corresponding to a resting, non-activated state at 3 dpi (D, inset in d). Arrows indicate cells shown in high magnification in the corresponding panels. Scale bar=50 μm in (A, B, C and D) and 20 μm (a, b, c and d). (E) Quantification of Iba-1^+^/BrdU^+^ cells at different time points after CCI or sham injury (mean±S.E.M., *n*=3–6). *P<0.05, **P<0.01, by two-tailed Student's *t* test comparing sham and CCI at each time point.

Microglial activation and proliferation are features of traumatic injury to the CNS (Loane & Byrnes, 2010). The microglial morphology in sham mice changed from ramified resident microglia to activated microglia at 1 dpi (Figure 2) in the pericontusional cortical region. Microglial morphology evolved further so that by 7 dpi, microglia had acquired an amoeboid macrophage-like morphology. As we were not able to differentiate between resident microglia and infiltrating macrophages with the Iba-1 antiserum, we assume that both microglia and macrophages comprise the BrdU^+^/Iba-1^+^ cell population. At 3 and 7 dpi, Iba-1^+^ cells are the predominant proliferating cell population in the CCI mice (Figures 2 and 6). The greatest number of BrdU^+^/Iba-1^+^ cells is present at 3 dpi in the perilesional area, in accordance with the maximum number of BrdU^+^ cells at this time point. To determine whether other cells of myeloid lineage were also proliferating at early time points, we stained sections with anti-CD11b. However, we did not detect BrdU^+^/CD11b^+^ cells that were not also Iba-1^+^ cells, suggesting that there were not many proliferating myeloid lineage cells other than Iba-1^+^ macrophages or microglia in the area adjacent to the lesion core at 1 or 3 dpi. In addition, we did not detect significant numbers of BrdU^+^/myeloperoxidase+ cells at 1 and 3 dpi (results not shown), suggesting the Cd11b^+^/BrdU^+^ cells were not of neutrophil origin. In sham-injured mice, Iba-1^+^/BrdU^+^ cells increased at 3 dpi, whereas only a few are present at 1 or 7 dpi.

### Astrocytic proliferation after CCI injury

**Figure 3:**
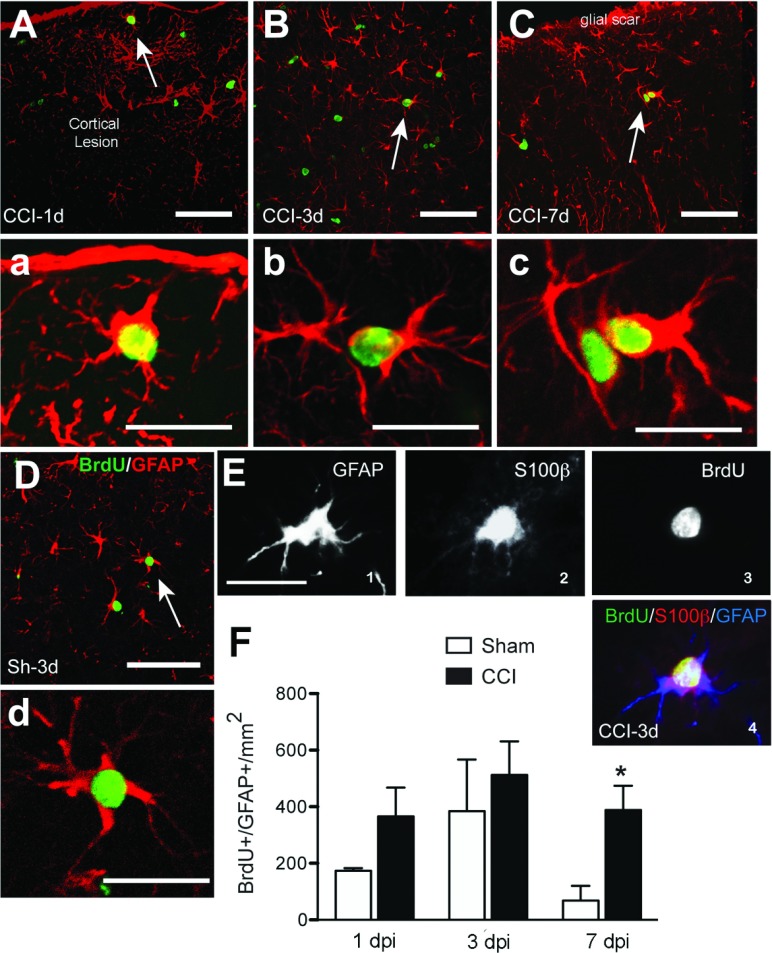
Astrocytic proliferation after CCI injury Co-localization of GFAP (red) and BrdU (green) after CCI or sham injury around the cortical lesion and in the corpus callosum at different time points. Proliferating astrocytes have the morphology of reactive astrocytes with hypertrophic cell body and processes. Arrows indicate cells shown at larger magnification in their corresponding panels. Some sections taken at 3 dpi were triple labeled for GFAP^+^/S100β^+^/BrdU^+^ (E1-4) confirming that dividing GFAP^+^ cells were astrocytes. Scale bar=50 μm (A, B, C and D) and 20 μm (a, b, c, d and E). (F) Quantification of GFAP^+^/BrdU^+^ cells at different time points after CCI or sham injury (mean±S.E.M., *n*=3–6). *P<0.05, **P<0.01, by two-tailed Student's *t* test comparing sham and CCI at each time point.

To determine whether astrocytes proliferated after injury, sections were double-labeled with antisera against BrdU and GFAP. The time course of proliferation was similar to that of Iba-1^+^ cells: proliferating astrocytes were detected as early as 1 dpi, but the greatest number were found at 3 dpi (Figure 3). By 7 dpi, there were fewer proliferating astrocytes, but as the total number of proliferating cells was lower at 7 dpi, the percentage of BrdU^+^ cells that were also GFAP^+^ was greater at 7 dpi than at 1 or 3 dpi. In the cortex, GFAP^+^/BrdU^+^ cells were preferentially localized close to the lesion, with a decreasing number further away from the lesion core (Figures 3A–3C). The number, size and morphology of the GFAP^+^ cells changed over time, with greater hypertrophy and thicker processes evident by 3 dpi (Figure 3), consistent with the known alterations in astrocyte morphology with activation. GFAP^+^/BrdU^+^ fibrous astrocytes were also detected in the corpus callosum by 1 dpi (results not shown), suggesting that astrocyte proliferation in the white matter may also be critical. To confirm the specificity of GFAP labeling, sections were also labeled with S100β to show that these cells were indeed astrocytes (Figure 3E). In sham-injured mice, GFAP^+^/BrdU^+^ cells followed a pattern similar to that in CCI mice at 1 and 3 dpi, although there are fewer doubly marked cells, and they are much reduced at 7 dpi.

### NG2^+^ cells are the most proliferative population at 24 h post-injury

**Figure 4:**
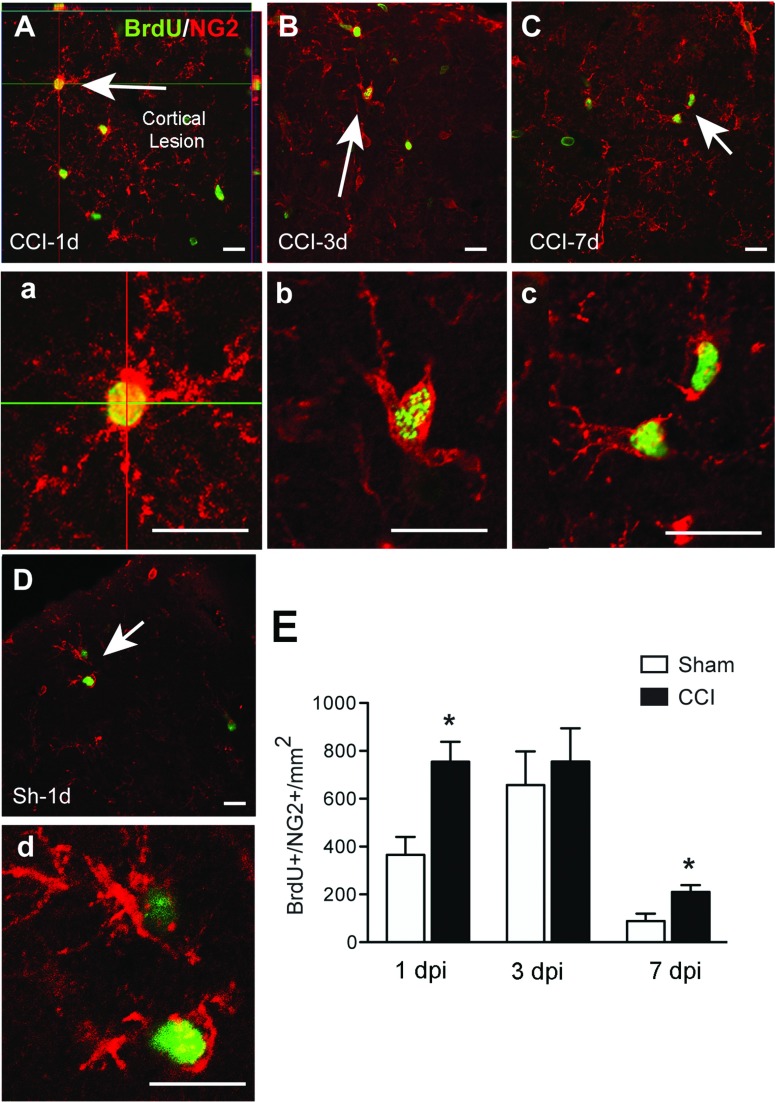
NG2 colocalizes with BrdU after CCI injury Confocal images show localization of proliferating NG2^+^ cells (NG2^+^, red; BrdU^+^, green) around the cortical site of injury at 1, 3 and 7 dpi. Proliferation of these cells was highest at 1 (A, a) and 3 dpi (B, b), although it was also high in sham brains (D, d). Proliferation of NG2^+^ cells decreased by 7 dpi (C, c). Arrows indicate cells shown at larger magnification in their corresponding panels. Scale bar=50 μm (A, B, C and D) and 20 μm (a, b, c and d). (E) Quantification of NG2^+^/BrdU^+^ cells at different time points after CCI or sham injury (mean±S.E.M., *n*=3–6). *P<0.05, by two-tailed Student's *t* test comparing sham and CCI at each time point.

NG2^+^ cells were observed proximal to the lesion border (Figures 4A–4C). NG2^+^ cells proliferated most rapidly after injury, with maximal BrdU incorporation at 1 dpi. Moreover, at 1 dpi, 47% of BrdU^+^ cells were NG2^+^. They were equally proliferative at 3 dpi, although as other cells increased their proliferation by this time point, the percentage of BrdU^+^ cells that were NG2^+^ decreased to 32%. By 7 dpi, proliferation of NG2^+^ cells had decreased significantly, to less than a quarter of their 1 dpi values (Figure 4E). Thus, NG2^+^ cells are the major proliferating cell type during the first few days after injury. In sham-injured mice, NG2^+^ cells were the major proliferative population at all three time points examined. This mimics the naive brain, where the NG2^+^ population are the most proliferative cell type in the brain parenchyma (Gensert & Goldman, 2001; Dawson et al., 2003). The increased NG2^+^ cell proliferation with craniotomy peaked at 3 dpi, with similar numbers of NG2^+^/BrdU^+^ in sham-injured mice (Figure 4). These results suggest that proliferation of NG2^+^ cells respond to general inflammatory signals generated by craniotomy in addition to the specific signals from the injured brain parenchyma.

### Proliferating neuroblasts at 3 days following CCI

**Figure 5:**
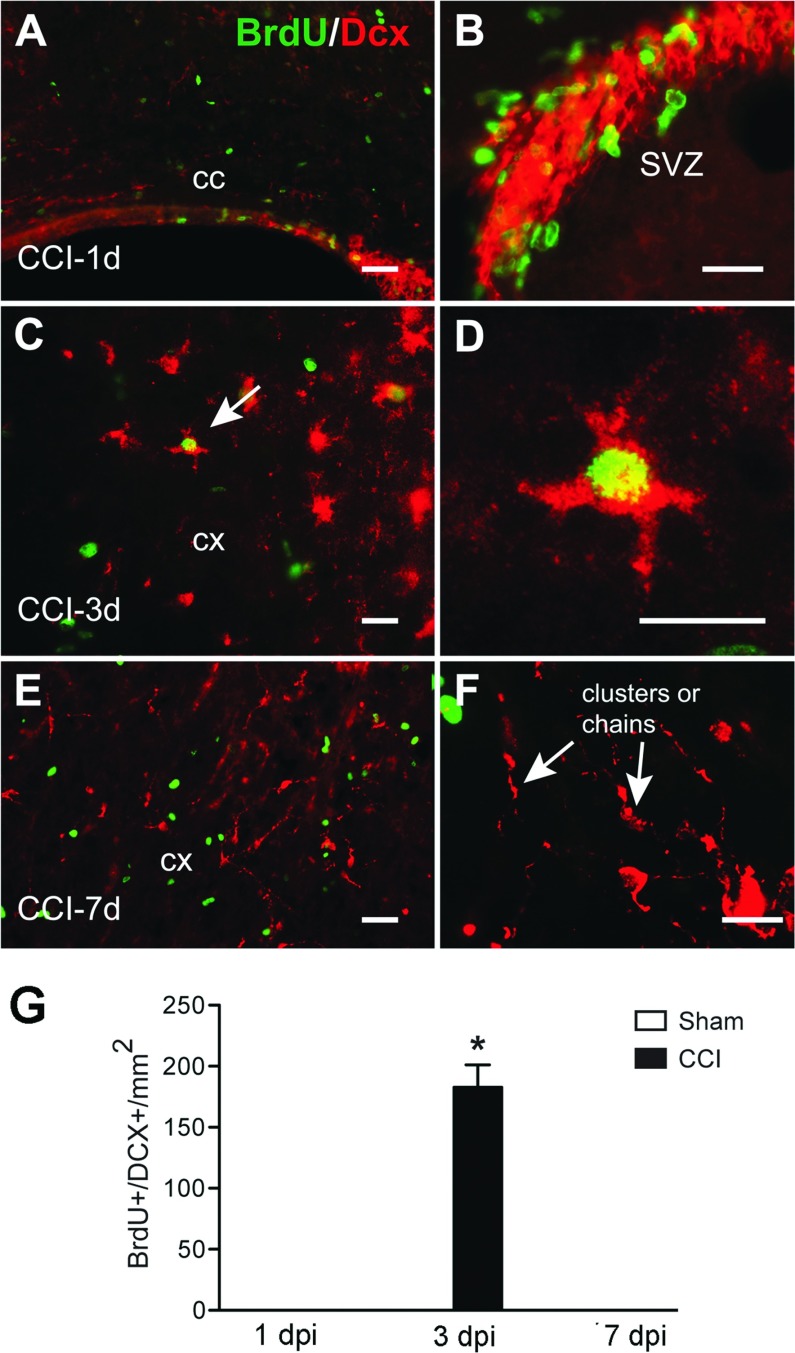
Proliferation of cortical DCX^+^ neuroblasts after CCI injury Confocal images show colocalization of both DCX^+^ (red) and BrdU^+^ (green) cells in the corpus callosum (cc) (A), in addition to the SVZ (B) at 1 dpi. DCX+/BrdU+ cells are seen in cortical areas at 3 dpi (C, cell indicated by arrow is shown in higher magnification in D). We observed no colocalization of BrdU and DCX at 7 dpi in the cortex (E); note the presence of clusters (or chains) of DCX^+^ cells (F) at 7 dpi. Scale bar=50 μm (A, C and E) and 20 μm (B, D and F). (G) Quantification of DCX^+^/BrdU^+^ cells after CCI or sham injury (mean±S.E.M., *n*=3–6). *P<0.05, by two-tailed Student's *t* test comparing sham and CCI at 3 dpi.

DCX is a marker uniquely expressed by immature neurons and migrating neuroblasts (Beach et al., 1999). It has been shown after injury that neuroblasts migrate from the SVZ to the lesion site (Parent, 2003). Concentrated labeling of DCX was observed in the SVZ at 1 and 3 dpi (results not shown). DCX^+^ cells were present in the cortex around the lesion only at 3 dpi (Figure 5). At 7 dpi DCX^+^ cells appear as clusters or chains, suggesting the morphology of migrating neurons. These cells were not labeled with BrdU. Double staining with BrdU and DCX demonstrated that only small population of proliferating neuroblasts was observed in the ipsilateral cortex at 3 dpi (Figure 5).

### Phenotype and fate of proliferating cells

**Figure 6:**
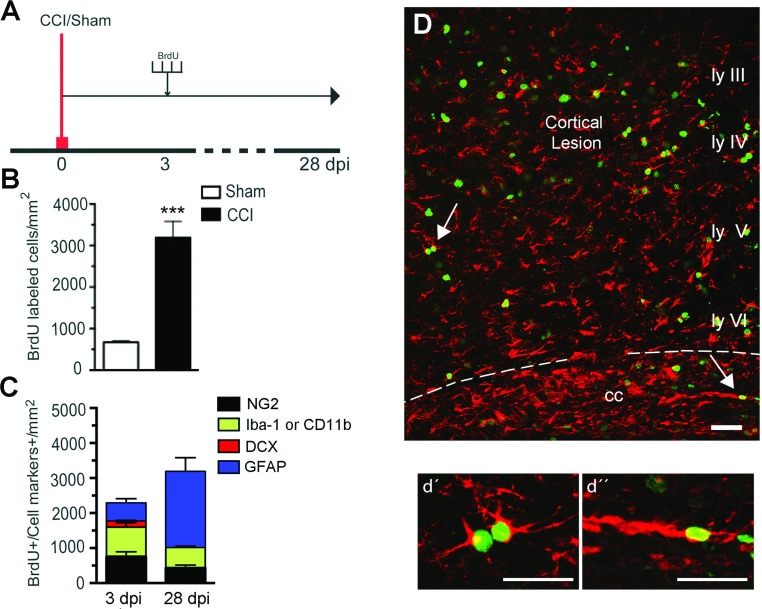
Fate of proliferating cells 4 weeks after CCI injury (A) Experimental design for BrdU injections. BrdU (100 mg/kg body weight) was injected at 3 dpi every 3 h for a total of four injections. One group of mice was killed at 3 dpi, and the second group at 28 dpi. (B) A significant increase in BrdU-labeled cells was found in the injured mice compared with sham mice at 28 dpi (***P<0.001). (C) Cell phenotype of BrdU^+^ cells in the ipsilateral injured cortex of mice killed at 3 or 28 dpi. At 28 dpi we observed more proliferating cells, most of which were BrdU^+^/GFAP^+^. The number of BrdU^+^ cells co-stained with Iba-1 or NG2 decreased, and no BrdU^+^/DCX^+^ cells were observed. (D) Confocal image showing colocalization of GFAP (red) and BrdU (green) across the cortical layers and corpus callosum at 28 dpi, and arrows point to proliferating astrocytes shown at high magnification in cortex (d’) and corpus callosum (d’’). Scale bar=50 μm (D) and 20 μm (d’ and d’). (mean±S.E.M., *n*=4).

To determine the fate of proliferating cells, selected mice were injected with BrdU at 3 dpi and euthanized at 28 dpi (Figure 6A). GFAP staining was still up-regulated around the lesion border at 28 dpi, with the GFAP staining extending to layer VI of the cortex and the corpus callosum (Figure 6D). Compared with mice that were injected at 3 dpi and killed 30 min after the last injection, there were more BrdU^+^ cells suggesting that cells continued to proliferate many days after CCI (Figure 6C). The phenotype of BrdU^+^ cells at 28 dpi was determined with co-labeling with the different cellular markers (NG2, Iba-1, GFAP, DCX). The number of GFAP^+^/BrdU^+^ cells at 28 dpi was nearly triple the number found in the mice killed at 3 dpi, and represented about 75% of all BrdU^+^ cells. At 28 dpi there were also BrdU^+^ cells that co-expressed either Iba-1 or NG2, although the number of these cells was lower than the GFAP^+^/BrdU^+^ cells (Figures 6C and 6D, d’,d’’). Thus the proportion of cell-specific BrdU^+^ cells labeled at 3 dpi was significantly different when the animals were killed immediately after injection, as opposed to 25 days later.

## DISCUSSION

Injury to the CNS is accompanied by a rapid reactive response that comprises an apparent increase in the number of astrocytes, OPCs and microglia. The question arises for each of these cell types as to whether the increase is due to proliferation or an increase in the expression of cell type-specific proteins by pre-existing cells.

The first reports of cell proliferation after CNS injury was by Adrian and Walker (1962) and Cavanagh (1970) who described an increase in ^3H^Thymidine-labeled cells in the cerebral cortex and spinal cord following a needle wound injury. Since that time, there have been many additional reports of cell proliferation in many different areas of the nervous system following traumatic injury (Dash et al., 2001; Braun et al., 2002; Chirumamilla et al., 2002; Rice et al., 2003). While many of these studies focused on the proliferation of neuronal stem cells and hippocampal neurogenesis in response to injury, the perilesional area of the cerebral cortex has been less studied (Kernie et al., 2001; Alonso, 2005; Ramaswamy et al., 2005b; Itoh et al., 2011). This area is important as the place to which endogenous neural progenitor cells may migrate after injury, and is the site for potential transplantation of stem cells. Thus, an understanding of the time course and pattern of proliferation of different cell types after injury provides essential information for developing strategies for cell-based therapies. In this publication, we used a saturation protocol for labeling cells with BrdU to label all proliferating cells, and have determined the temporal proliferation profile of glial cells soon after a CCI injury.

Various protocols have been used to label dividing cells after brain injury, initially using ^3H^Thymidine and more recently using BrdU or its analogs (Cavanagh, 1970; Taupin, 2007). Both labels are incorporated into cells that are actively synthesizing DNA. Because the residence time of these tracers in the brain is relatively short, repeated injections that encompass the cell cycle time, as we have done here, are necessary to label the entire proliferating fraction of all cell types; single injections will label different proportions of proliferating cells with different cell cycle times. Euthanasia soon after BrdU injection ensures that the number of labeled cells is not altered, either up or down, but accurately reflects cell proliferation (Hayes & Nowakowski, 2002). While some have reported incorporation as a result of DNA repair mechanisms, the protocol that we used, with multiple injections over a short period of time, labels all actively dividing cells (Gotz & Huttner, 2005) and thus the labeling is likely due to the recruitment of quiescent cells to become proliferative, rather than a result of the repair process.

Our results provide evidence that a moderate CCI brain injury induces cell proliferation within the area proximal to the injury as early as 1 dpi, with a peak at 3 dpi, and decreasing at 7 dpi. This pattern is similar to that described before in several studies in different models of brain injury, including CCI (Itoh et al., 2011), lateral fluid percussion injury (Urrea et al., 2007), cryogenic injury (Tatsumi et al., 2005) and stab wounds (Alonso, 2005). Furthermore, we identified these proliferating cells as primarily astrocytes, oligodendrocyte precursors, macrophages/microglia and only a few neuronal progenitors.

While the overall pattern of glial cell proliferation is similar for both microglia and macroglia, the particular time course of cell proliferation and the proportion of cells that are actively dividing differ for each of the cell types. Thus, NG2^+^ cells are the predominant proliferating cells at the earliest time, whereas the Iba-1^+^ macrophages/microglia predominate at later time points. This is quite similar to the time course and labeling index from other studies involving various types of cortical injury, although through the multiple injection protocol we employed, our study provides more conclusive evidence. Thus, Alonso (Alonso, 2005) found that at 3 h following a single injection of BrdU at 2 dpi, NG2^+^ cells were the largest proportion of the labeled cells (about 35%), followed by microglia (~20%), with very few labeled astrocytes.

NG2^+^ cells have traditionally been viewed as OPCs that can replenish mature oligodendrocytes in the adult brain (Nishiyama et al., 1999). However, these NG2 glia have many properties that are not consistent with a unique role as oligodendrocyte precursors. They are the major proliferating population in the adult brain, with equal distribution between gray and white matter (Nishiyama et al., 2002). After injury, NG2^+^ cells proliferate rapidly and can differentiate into oligodendrocytes given the right conditions, to replace oligodendrocytes lost after injury (Nishiyama et al., 2009). However, NG2^+^ cells can also differentiate into astrocytes and even neurons dependent on the type of injury, location of the cells and the environmental conditions (Aguirre et al., 2007; Zhu et al., 2008; Haselkorn et al., 2010). NG2^+^ cells are also critical for formation of the glial scar at later time points; secreting their own repertoire of chondroitin sulfate proteoglycans (including NG2 itself), which add to the inhibitory nature of the scar for neuronal regeneration (Butt et al., 2005; Tan et al., 2005).

Astrogliosis, characterized by an up-regulation of GFAP, is a cardinal response to brain injuries of all types, including CCI (Salman et al., 2004). Whether this is due to cell proliferation and/or activation of pre-existing astrocytes is still debatable. In this study, the number of proliferating astrocytes, as assessed by both GFAP and S100β immunoreactivity, was maximal at 3 dpi and occurred only in regions directly surrounding the cortical lesion. At 3 dpi, GFAP^+^/BrdU^+^ cells comprised approximately 25% of the total dividing cell population and approximately 20% of the GFAP^+^ cells (results not shown). This is similar to the 20% of double-labeled astrocytes found after lateral fluid percussion injury (Chirumamilla et al., 2002). Using a stab wound injury, Buffo et al. also reported approximately 15% of GFAP^+^ astrocytes were labeled with BrdU at 3 dpi in mice when BrdU was administered continuously in the drinking water (Buffo et al., 2008). In contrast, Alonso (Alonso, 2005) found only a few GFAP^+^/BrdU^+^ double-labeled cells after a single BrdU injection 2 days following a stab wound in rats. Thus, the timing and length of BrdU administration can make a considerable difference to the number of labeled cells. Overall, it would seem that proliferation does play a significant role in astrogliosis, and the amount of proliferation is dependent on the severity of the insult (Robel et al., 2011).

Whether cortical neuroblasts proliferate in response to injury is an open question. A small percentage of proliferative cells are DCX^+^ neuroblasts or immature migrating neurons, and they were only found at 3 dpi. Other studies have found no BrdU^+^ cells with neuronal markers after cortical injury (Tatsumi et al., 2005). It has been known for some time that there is increased neurogenesis after injury in the neurogenic niches of the SVZ and SGZ (subgranular zone) of the dendate gyrus and that DCX^+^ cells migrate toward the lesion area after stroke or TBI (Parent, 2003; Richardson et al., 2007; Zhang et al., 2007). Other studies have shown how CCI also results in the migration of BrdU-labeled neuroblasts to the injured cortex and toward the contralateral cortex, ipsilateral striatum and other subcortical structures (Chirumamilla et al., 2002; Ramaswamy et al., 2005a). However, the idea that neuronal precursors may originate locally in the cortex or even from leptomeningeal cells is gaining prominence (Nakagomi et al., 2012). We found an increase in DCX^+^ cells in the damaged cortex labeled with BrdU only at 3 dpi. As our protocol would only label cells that are synthesizing DNA at most 9.5 h before euthanasia, the compressed time course suggests that these were either locally derived proliferating cells that became DCX^+^ within the cortex, or DCX^+^ cells that migrated from the SVZ and were still able to proliferate once they reached the lesion area. DCX^+^/BrdU^+^ cells are a typical fate of progenitor cells.

To evaluate the cell fate of those cells that proliferate during the acute injury phase, we injected an experimental group of animals with BrdU at 3 dpi and euthanized them at 28 dpi. We found an increase in the number of BrdU^+^-labeled cells as compared with the animals euthanized at 3 dpi. The relatively modest increase in the number of BrdU^+^ cells 25 days after the injections is surprising given that we know all types of glial cells continue to proliferate until at least 7 dpi, and it is probable that the cells that proliferate at 3 and 7 dpi are not two distinct populations of cells. Our data therefore suggest the possibility that the more intensely proliferating cells lose their BrdU label by 28 dpi. Indeed it has been shown that the BrdU label is lost after three to four divisions (Hayes & Nowakowski, 2002). The proportion of GFAP^+^/BrdU^+^ cells was about 70% at 28 dpi as compared with 26% when killed at 3 dpi, suggesting that the NG2^+^ and/ or Iba-1^+^ cells are the faster proliferating cells that may lose their BrdU label, leaving astrocytes as the greater proportion of BrdU^+^ cells at 28 dpi. Alternatively, some NG2^+^ and Iba-1^+^ cells that proliferate at 3 dpi may differentiate into GFAP^+^ astrocytes by 28 dpi as suggested by Alonso (2005). A third possibility is that the non-astrocytic proliferating cells at 3 dpi may not survive as well as GFAP^+^/BrdU^+^ cells, altering the proportions of BrdU^+^ cell-types by 28 dpi.

Although sham mice have been considered throughout the literature as controls for TBI, the recent work has described a strong physiological response in sham-operated animals that is similar to a mild injury (Cole et al., 2011). This sham response is brief, and probably results from the increased inflammatory response to craniotomy that is sufficient to activate the signaling pathways to induce cellular proliferation (Lagraoui et al., 2012). By 7 dpi, proliferation in response to craniotomy alone was much reduced in agreement with the decreased inflammatory profile at this time point. We also found BrdU^+^ cells in the contralateral hemisphere at all the times studied (results not shown), similar to other reports (Kernie et al., 2001; Urrea et al., 2007) who proposed that these proliferative contralateral cells play a role in the cellular remodeling seen on the injured side.

In summary, we demonstrate a rapid and continuing bout of proliferation following CCI in the mouse that encompasses all the glial cell types. The timing of the proliferation of each cell type provides information as to the permissiveness of the perilesional area to support this proliferation. Because some of this proliferation is beneficial, while some other might have adverse effects, this knowledge can provide the framework for future work to manipulate these proliferative cells and thus engineer neural and behavioral recovery.

## Online data

Supplementary data
