# Supplementary material for: Temporal patterns of cortical proliferation of glial cell populations after traumatic brain injury in mice
Source: ASN Neuro. 2014 May 8;6(3):e00143. doi: 10.1042/AN20130034 (PMC4013687; doi:10.1042/AN20130034)
Supplement: Supplementary data [file an006e143add.pdf]

ASN NEURO RESEARCH ARTICLE

ASN NEURO 6(3):art:e00143.doi:10.1042/AN20130034

OPEN ACCESS

Temporal patterns of cortical proliferation of glial cell populations after traumatic brain injury in mice

Bala T.S. Susarla*†, Sonia Villapol*†, Jae-Hyuk Yi*‡, Herbert M. Geller‡ and Aviva J. Symes*†[1]

*Center for Neuroscience and Regenerative Medicine, Uniformed Services University of the Health Sciences, Bethesda, MD, U.S.A.

†Department of Pharmacology, Uniformed Services University of the Health Sciences, Bethesda, MD, U.S.A.

‡Developmental Neurobiology Section, Division of Intramural Research, National Heart, Lung, and Blood Institute, National Institutes of Health, Bethesda, MD, U.S.A.

SUPPLEMENTARY DATA

Table S1 Injury score

The injury score is calculated by determining the area of the cortical lesion as a percentage of the total area of the contralateral hemisphere, and represented with the scale (1–4) at different time points where 1 (<1 %), 2 (2–5 %), 3 (5–10 %) and 4 (>10 %). Values are showed as mean ± S.D., $n = 6$.

Days after injury	Injury score
1	2.75 ± 0.50
3	2.50 ± 0.54
7	3.66 ± 0.57
28	2.40 ± 0.54

[1] To whom correspondence should be addressed (email aviva.symes@usuhs.edu).

© 2014 The Author(s) This is an Open Access article distributed under the terms of the Creative Commons Attribution Licence (CC-BY) (http://creativecommons.org/licenses/by/3.0/) which permits unrestricted use, distribution and reproduction in any medium, provided the original work is properly cited.
